# Veterinarians’ perspectives on livestock diseases and antimicrobial use in Palestine

**DOI:** 10.14202/vetworld.2025.519-526

**Published:** 2025-02-27

**Authors:** Ibrahim M. Alzuheir

**Affiliations:** Department of Veterinary Medicine, Faculty of Veterinary Medicine and Agricultural Engineer, An-Najah National University, Nablus, Palestine

**Keywords:** antimicrobial resistance, antimicrobial stewardship, livestock diseases, Palestine, veterinary medicine

## Abstract

**Background and Aim:**

The livestock sector is a crucial component of Palestine’s agricultural economy, supporting food security and rural livelihoods. However, challenges such as infectious diseases, limited diagnostic resources, and antimicrobial misuse impact animal health and public safety. This study investigates veterinarians’ perspectives on disease prevalence and antimicrobial use in Palestinian livestock, providing the first comprehensive analysis of antimicrobial resistance (AMR) in veterinary practice in the region.

**Materials and Methods:**

A qualitative study was conducted using focus groups comprising 93 veterinarians from the West Bank, recruited through convenience and snowball sampling. A structured questionnaire collected data on disease prevalence and antimicrobial prescription patterns. Descriptive statistics and Chi-square tests were used to assess associations between demographic characteristics and veterinary practices.

**Results:**

Respiratory tract infections (RTIs) were the most frequently diagnosed disease (87.5%), followed by gastroenteritis (79.2%) and mastitis (75.0%). Veterinarians predominantly prescribed broad-spectrum antimicrobials, including penicillins (50.5%), tetracyclines (48.4%), and macrolides (46.2%). The use of antimicrobials classified as critically important for human medicine, such as quinolones (43.0%) and third-generation cephalosporins (46.2%), was notable. Some instances of banned antimicrobial use, such as chloramphenicol, were also reported.

**Conclusion:**

The findings highlight the reliance on empirical antimicrobial treatments and the widespread use of broad-spectrum and human-critical antimicrobials, raising concerns about AMR development. Improved antimicrobial stewardship, diagnostic capabilities, and regulatory frameworks are necessary to mitigate these risks. Policies promoting culture and sensitivity testing, along with targeted antimicrobial use, will enhance veterinary disease management and safeguard public health in Palestine.

## INTRODUCTION

The livestock sector in Palestine is pivotal to the agricultural economy, providing essential resources such as meat, milk, and wool to local communities [1]. This sector supports the livelihoods of numerous families and significantly contributes to regional food security and economic stability [2]. Veterinary services are crucial in this context, offering vital support for diagnosing and treating diseases, managing herd health, and ensuring the overall welfare of animals [3]. Despite its importance, the veterinary sector in Palestine faces significant challenges [4]. Limited resources, inadequate access to advanced diagnostic tools, and the need for effective disease management strategies impact the sector’s ability to maintain optimal livestock health [5]. The prevalence of various animal diseases and patterns of antimicrobial use are critical factors influencing the effectiveness of veterinary care [6].

Antimicrobial stewardship is particularly important in regions where livestock farming is central to the economy [7]. Effective antimicrobial use is essential for both animal health and public safety, especially in regions where overprescription and misuse of antimicrobials are prevalent [8]. The global rise of antimicrobial resistance (AMR) represents a significant threat to both human and animal health, with the misuse and overuse of antimicrobials in veterinary medicine being key drivers. Understanding the antimicrobials used by veterinarians in Palestine can provide valuable insights into current practices and highlight areas for improvement. This challenge is particularly crucial in regions like Palestine, where livestock farming is a central component of the economy [1, 2].

This study investigates veterinarians’ perspectives on disease prevalence and antimicrobial use in Palestinian livestock, providing the first comprehensive analysis of AMR in veterinary practice in the region. This study explores the landscape of veterinary services in Palestine by focusing on common livestock diseases and the practices associated with antimicrobial use. A comprehensive survey of veterinarians will gather data on disease prevalence and antimicrobial use, providing a detailed analysis of current practices. The findings will identify the most commonly used antimicrobial agents and their targeted diseases, forming the basis for improved disease management strategies and informed policy interventions. These findings not only support Palestine’s veterinary sector in Palestine and feed into the broader global discourse on AMR. These findings underscore the importance of integrating evidence-based policy reform, enhanced veterinary education, and public awareness to effectively combat AMR. Ultimately, this research highlights how localized efforts in regions like Palestine can align with international strategies to safeguard public health and sustainable agricultural systems.

## MATERIALS AND METHODS

### Ethical approval and Informed consent

This study received ethical review approval from the Palestinian Veterinarians Syndicate Ethics Committee (Ethics approval number: 16-5-24) on May 20, 2024. Participation was completely voluntary and informed verbal consent was obtained from all participants. Participant details were kept anonymous, and data confidentiality was rigorously maintained.

### Study period and location

The study was conducted in Palestine from May 25, 2024, to July 15, 2024. Data collection covered various veterinary practices across the North, Middle, and South regions of the West Bank.

### Questionnaire design

The questionnaire contained closed- and open-ended questions on the veterinarians’ sociodemographic data, most common diseases/cases associated with veterinary practice, and perceptions of antimicrobial use. The questions were developed through Google Forms (https://www.google.com/forms). The initial draft was created in English and later translated into Arabic. The questionnaire comprised three sections: (1) Sociodemographic characteristics, (2) open-ended questions about the most common diseases encountered in animal practice, and (3) multiple selection questions regarding antimicrobial use in practice. Open-ended and multiple-selection questions were chosen to balance prescriptive insights with structured data, ensuring a comprehensive exploration of participant perspectives. In this survey, we requested reporting on the diseases most commonly encountered in different veterinary practices (large animals, small animals, equine, poultry, and veterinarians with mixed practices). We did not ask the veterinarians whether they had reached the diagnosis empirically or whether they had obtained samples for laboratory confirmation to confirm the etiology. Nevertheless, we proceeded with data analysis for all diseases reported by the participants, as all were treated with antimicrobials.

Two expert researchers reviewed the draft for content validity and ambiguity. A pilot test was conducted with 5 veterinarians to assess the questionnaire duration, clarity, and sequence.

### Study population and sampling procedure

The study targeted veterinarians in Palestine. Instead of relying solely on a list of registered veterinarians from the Palestinian Veterinarians Association, a snowball sampling method was used. The initial participants were identified through professional contacts and social media groups, including veterinarians’ professional networks. The initial participants were then asked to refer to other veterinarians in their network that were eligible for participation. This method enabled broader participation by leveraging personal and professional connections, ensuring a more diverse respondent base. The questionnaire was distributed online through Google Forms from May 25, 2024, to July 15, 2024. Participants were encouraged to share the questionnaire link with other veterinarians; they knew how to enhance response rates, following a snowball sampling approach. The method was selected for its practicality in reaching veterinarians who may not be listed in formal registries but are active in practice. The tapping into professional networks ensured a broader and more diverse participation base, which was essential for gaining a comprehensive understanding of veterinary practices in Palestine. Despite its limitations, the method was deemed appropriate for obtaining valuable preliminary insights when other sampling options were not feasible.

### Statistical analysis

All collected data were reviewed for completeness and consistency before analysis. The responses were manually checked and coded in Microsoft^®^ Office Excel 365 (Microsoft Corp., Washington, USA), followed by statistical processing using IBM Statistical Package for the Social Sciences Statistics version 21.0 (SPSS Inc., Chicago, IL, USA).

Descriptive statistics were employed to summarize the demographic characteristics of participants and the frequency distribution of diseases and antimicrobial use. Categorical variables, such as disease prevalence and antimicrobial prescription patterns, were expressed as frequencies and percentages.

Associations between veterinarians’ demographic characteristics (age, gender, practice type, and years of experience) and their reported antimicrobial prescription patterns were assessed using the Chi-square (χ^2^) test. The statistical significance threshold was set at p < 0.05.

For disease prevalence, comparisons between different veterinary practice types (farm animals, small animals, equine, poultry, and mixed practices) were conducted using cross-tabulations and χ^2^ tests. In addition, the correlation between commonly prescribed antimicrobials and diagnosed diseases was analyzed to identify trends in empirical treatment practices.

To evaluate potential regional variations in disease prevalence and antimicrobial use across the North, Middle, and South regions of the West Bank, we conducted one-way analysis of variance for normally distributed data and Kruskal–Wallis tests for non-normally distributed data. *Post hoc* pairwise comparisons were performed using Bonferroni adjustments when significant differences were detected.

All statistical tests were two-tailed, and results were considered statistically significant at p < 0.05.

## RESULTS

A total of 93 veterinarians participated in the survey using the snowball convenient sampling method. The majority of participants were male (91.4%), with a smaller female representation (8.6%). Most participants were under 30 years old (44.1%) or between 31 and 40 years old (47.3%). Geographically, the sample is evenly distributed among the North (30.1%), Middle (31.2%), and South (38.7%) regions of the West Bank and Gaza. Veterinary practice largely focuses on mixed animals (48.4%), with notable representation in farm animals (25.8%) and smaller numbers in small animals, poultry, and equine practices. Experience levels vary, with the majority having <10 years (59.1%) or between 11 and 20 years (35.5%) in the field (Table 1).

**Table 1:** Demographic characteristics of surveyed veterinarians in Palestine, 2024.

Variable	Number of veterinarians categories, n (%)	Confidence interval (95%)
Gender		
Male	85 (91.4)	84.9, 86.8
Female	8 (8.6)	3.2, 15.1
Age (years)		
<30	41 (44.1)	34.4, 53.8
31–40	44 (47.3)	36.6, 57.0
41–50	4 (4.3)	1.1, 8.6
>50	4 (4.3)	1.1, 8.6
District		
North	28 (30.1)	21.5, 39.8
Middle	29 (31.2)	21.5, 40.9
South	36 (38.7)	29.0, 48.4
Veterinary practice		
Farm animals	24 (25.8)	17.2, 35.3
Small animals	14 (15.1)	7.6, 22.6
Equine	4 (4.3)	1.1, 8.6
Poultry	6 (6.5)	2.2, 11.8
Mix	45 (48.5)	37.6, 59.1
Experience (years)		
<10	55 (59.1)	49.5, 68.8
10–20	33 (35.5)	26.9, 45.2
21–30	4 (4.3)	1.1, 8.6
>31	1 (1.1)	0.0, 3.2

### Most prevalent diseases

**Figure 1:**
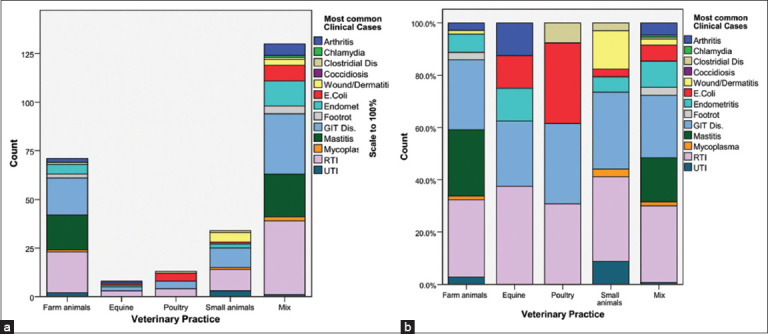
Distribution of clinical cases across different veterinary practices. (a) The count of most common clinical cases reported in different veterinary practices, including farm animals, equine, poultry, small animals, and mixed practices. (b) The same data scaled to 100%, highlighting the proportion of each clinical case within veterinary practice. Common clinical cases include arthritis, chlamydia, clostridial diseases, coccidiosis, wound/dermatitis, *E. coli* infections, endometritis, footrot, gastrointestinal diseases, mastitis, mycoplasma, RTI, and UTI. RTI=Respiratory tract infections, UTI=Urinary tract infections, *E. coli=Escherichia coli*.

The data revealed the distribution of various diseases across different types of veterinary practices (Figure 1). Based on veterinarians’ reports, respiratory tract infections (RTIs) were the most prevalent in farm animals, accounting for 87.5% of cases, followed by gastroenteritis (79.2%) and mastitis (75.0%) in the investigated regions. Footrot and arthritis were less common, representing 8.3% of cases. *Escherichia coli* and urinary infections were also reported at 8.3%, whereas wound/dermatitis and mycoplasma each accounted for 4.2% of infections. In small animal practice, RTIs dominate, accounting for 78.6% of cases, with gastroenteritis accounting for 71.4%. Wound/dermatitis was reported in 35.7% of cases, and urinary infections accounted for 21.4%. *E. coli*, Mycoplasma, and Clostridia each represented 7.1% of the cases, whereas endometritis was found in 14.3%. RTIs were most common in equine practice, accounting for 75.0% of cases, followed by gastroenteritis and endometritis, each accounting for 50.0% of cases. *E. coli* and arthritis each accounted for 25.0% of the cases, with no other diseases reported. In poultry practices, *E. coli*, RTIs, and gastroenteritis represent 66.7% of all cases, whereas Clostridia account for 16.7%. No other diseases have been reported in poultry. Mixed practices were associated with high frequencies of RTIs (84.4%), gastroenteritis (68.9%), and mastitis (48.9%). Endometritis occurred in 28.9% of the cases, with 17.8% involving *E. coli* and 8.9% involving foot rot. Arthritis occurred in 13.3% of cases, whereas wound/dermatitis, mycoplasma, and clostridia each represent approximately 6%–7% of cases. Notably, no significant differences in disease distribution were observed across the investigated regions.

### Antimicrobials use

**Figure 2:**
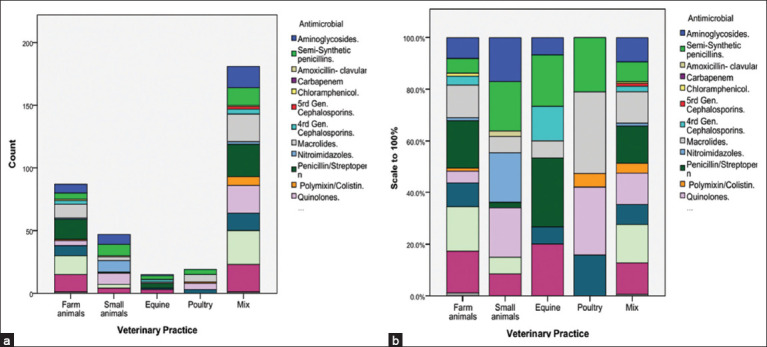
Antimicrobial use across different veterinary practices. (a) Antimicrobial usage in different veterinary practices, including farm animals, small animals, equine, poultry, and mixed practices. (b) The antimicrobial usage data scaled to 100%, showing the proportion of each type of antimicrobial used in veterinary practice. Antimicrobials include aminoglycosides, semi-synthetic penicillins, amoxicillin-clavulanic acid, carbapenem, chloramphenicol, fifth and fourth-generation cephalosporins, macrolides, nitroimidazoles, penicillin/streptomycin, polymyxin/colistin, and quinolones.

The results revealed a wide range of antimicrobials used in veterinary practice in the livestock sector in Palestine (Figure 2). Penicillin/streptopenicillin had the highest usage, with 47 responses, accounting for 13.5% of the total responses, and was used in 50.5% of the cases. Tetracycline/oxytetracycline also showed high usage, with 45 responses, accounting for 12.9% of the total responses and 48.4% of the cases. Macrolides and third-generation cephalosporins each had 43 responses, representing 12.3% of the total responses, and were used in 46.2% of the cases. There was moderate use of quinolones and aminoglycosides, where quinolones (enrofloxacin, ciprofloxacin, norfloxacin) had 40 responses, accounting for 11.5% of the total responses and were used in 43.0% of the cases. Aminoglycosides (gentamicin) had 33 responses, accounting for 9.5% of the total responses and 35.5% of the cases. Intermediate usage of sulfonamides and nitroimidazoles: Sulfonamides had 26 responses, accounting for 7.4% of the total responses, and were used in 28.0% of the cases. Nitroimidazoles (metronidazole) had 12 responses, accounting for 3.4% of the total responses, and were used in 12.9% of the cases. Certain antimicrobials with low usage include fourth-generation cephalosporins (cefepime, cefquinome) and polymyxin/colistin, each with 9 responses, accounting for 2.6% of the total responses and being used in 9.7% of the cases. Five-generation cephalosporins (ceftaroline), glycopeptides (vancomycin), and chloramphenicol had very low usage, with only 2 responses each, accounting for 0.6% of the total responses and 2.2% of the cases. Amoxicillin-clavulanic had the lowest usage, with only 1 response, representing 0.3% of the total responses and 1.1% of the cases.

### Common diseases and the antimicrobial usage

**Figure 3:**
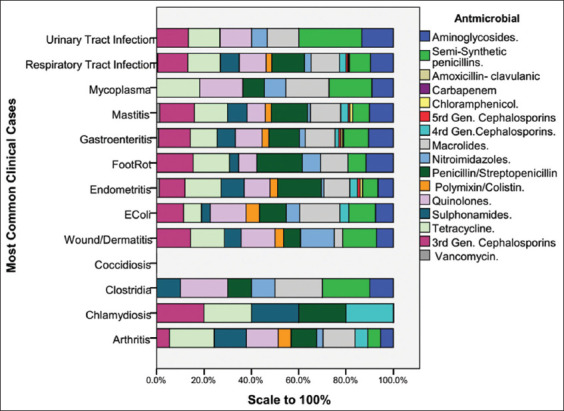
Antimicrobial usage by clinical cases. This figure shows the distribution of different antimicrobials used for various clinical cases, including arthritis, chlamydia, clostridial diseases, coccidiosis, wound/dermatitis, *E. coli* infections, endometritis, footrot, gastrointestinal diseases, mastitis, mycoplasma, RTI, and UTI. Antimicrobials are categorized into aminoglycosides, semi-synthetic penicillins, amoxicillin-clavulanic acid, carbapenem, chloramphenicol, fifth and fourth-generation cephalosporins, macrolides, nitroimidazoles, penicillin/streptomycin, polymyxin/colistin, quinolones, sulfonamides, tetracyclines, third-generation cephalosporins, and vancomycin. RTI=Respiratory tract infections, UTI=Urinary tract infections, *E. coli=Escherichia coli*.

The data presented show the distributions of various diseases and their associations with different antimicrobial agents (Figure 3). *E. coli* infections frequently involve quinolones (57.1%) and macrolides (64.3%), RTIs rely heavily on semisynthetic penicillins (51.9%) and tetracyclines (51.9%), and gastroenteritis shows considerable use of semisynthetic penicillin (48.5%). Footrot is primarily treated with semisynthetic penicillins (83.3%) and quinolones (50.0%). RTIs dominated the dataset, with high percentages of patients receiving treatment with semisynthetic penicillins (51.9%), tetracyclines (51.9%), and macrolides (45.5%). Gastroenteritis is also common, with considerable reliance on semisynthetic penicillin (48.5%) and quinolones (42.4%). Endometritis has diverse antimicrobial uses, notably with semisynthetic penicillin (81.0%) and tetracyclines (66.7%). Mastitis treatment frequently involved semisynthetic penicillin (60.0%) and tetracyclines (55.0%). Urinary infections are less prevalent but still involve the use of semisynthetic penicillin (66.7%). Patients with wound/dermatitis commonly use semisynthetic penicillin (44.4%) and tetracyclines (44.4%). *Chlamydia, Mycoplasma, Clostridia*, and arthritis require various but less common antimicrobial treatments. The overall data highlight a high prevalence of RTIs and gastroenteritis, with significant usage of semisynthetic penicillin, quinolones, and tetracyclines across multiple diseases.

## DISCUSSION

Veterinarians in Palestine have reported numerous cases and disease conditions in livestock, along with the antimicrobials used for treatment. This study is the first to investigate antimicrobial use in livestock in Palestine, highlighting the significant gap between regional and global data. While some studies on AMR exist globally, data from the Middle East, including Palestine, remain scarce, underscoring the urgency for regional research and action [9, 10]. This study is the first to report on the use of antimicrobials in livestock in Palestine. Our findings revealed that most participants were male and aged 30–40 years, reflecting a significant underrepresentation of females in the veterinary field in Palestine [11]. This gender imbalance contrasts with trends in countries such as Canada, the United States, and Portugal, where women are predicted to represent most of the veterinary profession [10, 12]. This difference highlights a regional variation in gender representation and suggests that the veterinary profession in our area may benefit from initiatives aimed at encouraging female participation and addressing any barriers to their entry into and advancement in the field. The gender imbalance in the field of veterinary medicine stems from traditional stereotypes, high costs, and challenging work conditions. Addressing these barriers through targeted initiatives, such as improving work-life balance, providing financial support for education, and fostering inclusive workplaces, could diversify and strengthen the profession [13].

A wide variety of diseases treated by veterinarians in livestock have been reported. Among the 256 diseases reported by veterinarians, respiratory diseases are the most frequently diagnosed diseases in all animal practices and frequently lead to the prescription of systemic antimicrobial agents. No differences in disease distribution were observed across the investigated regions, indicating a uniform prevalence of veterinary diseases. These findings are consistent with those of regions that reported RTIs as the most prevalent infections across veterinary practices [14]. Respiratory infection involves many etiological agents, including viruses, bacteria, parasites, and others, leading veterinarians to rely on broad-spectrum antimicrobials such as penicillin/streptopenicillin, quinolones, third-generation cephalosporins, and tetracycline [15]. These results are similar to those reported in Europe [7]. The preference for broad-spectrum agents reflects global challenges and raises concerns about the development of resistance. Although effective for a range of infections, their overuse underscores the need for enhanced diagnostic capabilities and the promotion of narrow-spectrum antimicrobials whenever possible [16]. The lower use of fifth-generation cephalosporins and glycopeptides may be attributed to their reserved application for severe infections or concerns regarding resistance and side effects. However, food animals should not be treated with “critically important” or “last line” antimicrobials for humans (namely, glycopeptides, colistin, and carbapenems) [8].

Palestine’s lack of regulations on antimicrobial use in veterinary practice intensifies the risk of resistance. The use of banned substances like chloramphenicol in food-producing animals and the absence of surveillance programs highlight critical gaps [17]. Implementing treatment guidelines tailored to local disease profiles, alongside robust surveillance programs, is essential. Globally, countries with stringent AMR policies, such as the Netherlands and Denmark, have successfully reduced antimicrobial use in livestock without compromising animal health, offering valuable models for Palestine and neighboring countries [18].

A better understanding of pathophysiology, pharmacodynamics, pharmacokinetics and better diagnostic procedures could reduce the inappropriate use of antimicrobials in animals [19]. Implementing culture and sensitivity tests for targeted therapy and regularly monitoring resistance patterns will help optimize treatment and safeguard both animal and public health.

Gastroenteritis and mastitis are closely followed by RTIs [20]. Endometritis and arthritis occur less frequently, whereas diseases such as *E. coli* infections, foot rot, urinary infections, wound/dermatitis, mycoplasma, clostridia, and chlamydia are observed even less often [21]. The observed variation in disease prevalence underscores the need for customized health management strategies [22]. Similar trends in antimicrobial use for RTIs were observed under these disease conditions.

The current use of antimicrobials in veterinary practice by veterinarians in Palestine raises concerns about potential resistance development. To address this issue, regular monitoring and evaluation of antimicrobial use is essential to identify trends and improve practices aimed at effective treatments and minimizing resistance risks. Promoting antimicrobial stewardship by encouraging the use of narrow-spectrum antimicrobials and adhering to established guidelines is crucial [16]. Further research is required to assess the effectiveness of various treatment strategies. The implementation of targeted therapy through culture and sensitivity tests can increase the precision of antimicrobial treatment. In addition, systematic monitoring and reporting of antimicrobial use and resistance patterns will help to adapt treatment protocols effectively. These strategies will improve antimicrobial use, reduce resistance, and enhance treatment outcomes. In the Eastern Mediterranean Region, efforts such as restricted antimicrobial access, national action plans, and awareness campaigns have demonstrated progress, but significant challenges remain. Strengthening collaboration through AMS and the One Health approach is critical for effectively combating AMR [23].

## CONCLUSION

This study provides the first comprehensive assessment of veterinarians’ perspectives on livestock diseases and antimicrobial use in Palestine, shedding light on critical gaps in antimicrobial stewardship and disease management. The findings reveal that RTIs (87.5%), gastroenteritis (79.2%), and mastitis (75.0%) are the most prevalent diseases across veterinary practices. Empirical antimicrobial use remains widespread, with penicillins (50.5%), tetracyclines (48.4%), and macrolides (46.2%) being the most commonly prescribed classes. Notably, the frequent use of quinolones (43.0%), third-generation cephalosporins (46.2%), and instances of banned antimicrobial use highlight concerns regarding AMR.

This study has several strengths. It is the first regional assessment of AMR in veterinary medicine, filling a crucial knowledge gap. The structured survey method ensured a broad representation of veterinarians across different livestock sectors, providing empirical insights for policy interventions.

Despite these strengths, some limitations should be acknowledged. The use of convenience and snowball sampling may introduce selection bias, limiting the generalizability of findings. In addition, disease prevalence was based on veterinarians’ self-reported diagnoses without laboratory confirmation, which may lead to potential misclassification of cases. The study’s cross-sectional design does not capture temporal trends in antimicrobial use or resistance patterns, making it difficult to assess long-term changes. Furthermore, factors influencing veterinarians’ prescription decisions, such as availability of antimicrobials, economic constraints, and farmer compliance, were not explored in detail.

Future research should focus on longitudinal surveillance of AMR trends to monitor the emergence of resistant pathogens in livestock. The implementation of culture and sensitivity testing in veterinary practice is necessary to reduce reliance on empirical treatments. Further studies should evaluate the effectiveness of antimicrobial stewardship programs and assess the impact of training and policy interventions on prescribing behaviors. A One Health approach, linking veterinary antimicrobial use with human and environmental health outcomes, is essential for developing holistic AMR mitigation strategies.

Addressing AMR in Palestine’s veterinary sector requires evidence-based policies, improved diagnostic capabilities, and enhanced veterinary education to promote responsible antimicrobial use. The study underscores the urgency of regulatory frameworks, public health collaboration, and integration into global AMR mitigation efforts to safeguard both animal and human health.

## AUTHOR’S CONTRIBUTIONS

IMA: Conceptualized and designed the study, collected and analyzed the data, and drafted, revised, and approved the final manuscript.
